# Concurrent immunotherapy and re‐irradiation utilizing stereotactic body radiotherapy for recurrent high‐grade gliomas

**DOI:** 10.1002/cnr2.1788

**Published:** 2023-02-07

**Authors:** Sean S. Mahase, Michelle Roytman, Diana Roth O'Brien, Jana Ivanidze, Theodore H. Schwartz, Susan C. Pannullo, Rohan Ramakrishna, Rajiv S. Magge, Nicholas Williams, Howard A. Fine, Gloria Chia‐Yi Chiang, Jonathan P. S. Knisely

**Affiliations:** ^1^ Department of Radiation Oncology NewYork Presbyterian–Weill Cornell Medicine New York New York USA; ^2^ Department of Radiation Oncology Penn State Cancer Institute Hershey Pennsylvania USA; ^3^ Department of Radiology NewYork Presbyterian–Weill Cornell Medicine New York New York USA; ^4^ Department of Neurosurgery NewYork Presbyterian–Weill Cornell Medicine New York New York USA; ^5^ Department of Neurology NewYork Presbyterian–Weill Cornell Medicine New York New York USA; ^6^ Department of Healthcare Policy & Research, Division of Biostatistics and Epidemiology Weill Cornell Medicine New York New York USA

**Keywords:** immune checkpoint inhibitor, nivolumab, pembrolizumab, recurrent high‐grade glioma, stereotactic body radiotherapy

## Abstract

**Background:**

Clinical trials evaluating immune checkpoint inhibition (ICI) in recurrent high‐grade gliomas (rHGG) report 7%–20% 6‐month progression‐free survival (PFS), while re‐irradiation demonstrates 28%–39% 6‐month PFS.

**Aims:**

We evaluate outcomes of patients treated with ICI and concurrent re‐irradiation utilizing stereotactic body radiotherapy/fractionated stereotactic radiosurgery (SBRT) compared to ICI monotherapy.

**Methods and Results:**

Patients ≥18‐years‐old with rHGG (WHO grade III and IV) receiving ICI + SBRT or ICI monotherapy between January 1, 2016 and January 1, 2019 were included. Adverse events, 6‐month PFS and overall survival (OS) were assessed. Log‐rank tests were used to evaluate PFS and OS. Histogram analyses of apparent diffusion coefficient maps and dynamic contrast‐enhanced magnetic resonance perfusion metrics were performed. Twenty‐one patients with rHGG (ICI + SBRT: 16; ICI: 5) were included. The ICI + SBRT and ICI groups received a mean 7.25 and 6.2 ICI cycles, respectively. There were five grade 1, one grade 2 and no grade 3–5 AEs in the ICI + SBRT group, and four grade 1 and no grade 2–5 AEs in the ICI group. Median PFS was 2.85 and 1 month for the ICI + SBRT and ICI groups; median OS was 7 and 6 months among ICI + SBRT and ICI groups, respectively. There were significant differences in pre and posttreatment tumor volume in the cohort (12.35 vs. 20.51; *p* = .03), but not between treatment groups.

**Conclusions:**

In this heavily pretreated cohort, ICI with re‐irradiation utilizing SBRT was well tolerated. Prospective studies are warranted to evaluate potential therapeutic benefits to re‐irradiation with ICI + SBRT in rHGG.

## 1 INTRODUCTION

High‐grade gliomas (HGG), including glioblastoma (GBM) and anaplastic astrocytomas, are associated with a poor prognosis and quality of life.^1^, ^2^, ^3^, ^4^, ^5^ The majority of HGGs recur, at which point treatment options include re‐resection, re‐irradiation, bevacizumab, ‘off‐label’ chemotherapy, tumor‐treating fields, or clinical trial enrollment.^6^, ^7^, ^8^ Unfortunately, recurrent high‐grade glioma (rHGG) trials historically produce high failure rates,^9^, ^10^, ^11^ necessitating novel therapeutic approaches.

Immune checkpoint inhibitors (ICIs) provided impressive results in melanoma, nonsmall cell lung cancer,^12^ and in untreated brain metastases secondary to these malignancies.^13^ While several clinical trials evaluating ICI in HGGs are ongoing, results are disappointing to date. Prior studies evaluating salvage re‐irradiation report 28% to 39% 6‐month progression‐free survival (PFS) rates, with a 26% median 1‐year overall survival (OS) rate.^11^, ^14^, ^15^, ^16^, ^17^, ^18^ Radiotherapy (RT) may improve ICI efficacy through several mechanisms, including altering tumor cell surface proteins, and enhancing the availability and assortment of intracellular peptide pools. These effects, in conjunction with inducing MHC class I expression, provides a larger repertoire of antigenic targets to elicit an immune response. RT induces major histocompatibility complex (MHC) class I expression through upregulating interferon‐γ, promoting T cell recruitment,^19^ conferring increased survival compared with either modality alone in mouse models.^20^ Additionally, stereotactic body radiotherapy / fractionated stereotactic radiosurgery (SBRT), entailing conformally delivering higher RT doses in fewer treatments, may be preferable over conventional RT delivered over several weeks with regard to augmenting immune responses,^21^ while minimizing the impact on circulating lymphocytes.

Combining ICI with concurrent RT may increase the therapeutic ratio in rHGG, however, there are no prospective studies evaluating toxicities and outcomes of concurrent re‐irradiation with ICI + SBRT. We report treatment‐related adverse events (AE) in patients with rHGG treated with concurrent ICI + SBRT. PFS, OS and changes in tumor volume and perfusion characteristics after treatment were also evaluated.

## 2 METHODS

### 2.1 Patient selection

This study was an institutional review board approved retrospective review (19–07020426) of patients age > 18 at the time of rHGG diagnosis (WHO grade IV GBM or WHO grade III anaplastic astrocytoma) treated with concurrent ICI + SBRT or ICI monotherapy between January 1, 2016 and January 1, 2019. All studies involving human participants were in accordance with the ethical standards of the institutional research committee and with the 1964 Helsinki Declaration and its later amendments or comparable ethical standards. No identifiable personal health information is reported in this analysis. Demographic data, tumor pathological characteristics and profiling from available Foundation studies, radiology variables (tumor size, perfusion/diffusion metrics, and RT necrosis), prior treatments, AE's attributable to treatment, PFS and OS following concurrent ICI + SBRT were collected. Survival data was obtained from available medical records. In total, 356 patients with rHGG were evaluated from January 2016 to January 2019 (Supplementary Figure 1). Patients were excluded if they did not receive ICI monotherapy, ICI concurrently with SBRT (n = 333), or if they had outside imaging during treatment that was not available for analysis (*n* = 2), leaving 21 patients for analysis.

### 2.2 Treatment

Patients treated with nivolumab received intravenous infusions of 3 mg/kg, administered on days 1 and 14 of a 28‐day cycle. Patients treated with pembrolizumab received intravenous infusions of 2 mg/kg, administered on days 1 and 21 of a 21‐day cycle. A subsequent ICI cycle was delayed until recovery of grade 3 or higher hematologic or grade 2 or higher nonhematologic toxicities.

RT planning volumes were created by a radiation oncologist and neurosurgeon. Dose and fractionation were determined on the basis of lesion size, prior radiotherapy, and meeting dose constraints for adjacent organs at risk. For treatment planning, high‐resolution 1 mm magnetic resonance T1 sequences with contrast were rigidly fused to CT simulation scans. Treatment was delivered using volumetric modulated arc therapy (VMAT) of 3–4 noncoplanar arcs and either 6× or 10× flattening filter‐free beams ensuring 95% of the planning target volume received the prescribed dose. RT plans were generated using Eclipse v15.6 (Varian Medical Systems, Palo Alto, CA) with AAA or AcurosXB planning algorithms. Organ at risk constraints for SBRT plans adhered to TG101 guidelines. RT was delivered on a Novalis (BrainLab, Munich, Germany) Truebeam STX linac (Varian Medical Systems, Palo Alto, CA), with multileaf collimator leaf width of 2.5 mm.

### 2.3 Imaging

All patients underwent brain MRI on 1.5 or 3 Tesla systems (Skyra, Aera, Biograph mMR, Siemens Healthcare; Discovery 750 w, Signa HDxt, GE Healthcare, Milwaukee, WI), pre and posttreatment per institutional standards as previously described.^22^

Olea Medical 3.0 software (La Ciotat, France) was used for DCE perfusion MRI processing and histogram analysis. The volumes‐of‐interest encompassing all voxels with enhancing tumor, pre and posttreatment were including in histogram analysis to produce blood–brain barrier permeability metrics, including median, mean, and 90% of the plasma volume (Vp) and volume transfer constant (Ktrans). Diffusion metrics, including median, mean, and 10% of the ADC were also evaluated. All values were normalized utilizing the contralateral normal white matter.

### 2.4 Evaluation

Routine testing included weekly laboratory tests including complete blood counts and basic metabolic panel, examinations at every clinical visit, and contrast‐enhanced brain MRI every 4 weeks.

Neuroradiologic response following treatment was determined by response assessment in neuro‐oncology (RANO) criteria.^23^ Complete response (CR) entailed disappearance of all contrast‐ and noncontrast enhancing tumor on consecutive MRIs with minimal 1‐month interval, and in the absence of corticosteroids. Partial response (PR) was defined by >50% reduction in tumor size derived by the sum of cross‐sectional radii on consecutive MRI scans of minimal 1‐month interval, and in the presence of stable or decreased corticosteroid dose. Progressive disease (PD) was defined as greater than 25% increase in tumor size or interval development of new lesions. Stable disease (SD) entailed all other scenarios and required confirmation MRI following best reported response.^23^ Patients continued ICI until they progressed or developed unacceptable AE, at which time patients either had bevacizumab added to their regimen or discontinued ICI.

AE were retrospectively determined for all patients and tabulated using Common Terminology Criteria for Adverse Events version 5.0. PFS and OS were defined as time from the day one of ICI until disease progression and death, respectively.

### 2.5 Statistical analysis

Descriptive statistics (including mean, standard deviation, median, interquartile range, frequency, and percent) were used to characterize the study sample (i.e., demographics, tumor profiling, clinical outcomes, adverse effects, and radiographic factors). Kaplan–Meier survival analysis descriptively assessed PFS and OS. With a sample size of 21 patients, two‐sided 95% confidence intervals for PFS/OS at defined time points of interest (i.e., six‐months, etc.) were constructed to be within ±22.8% of the observed survival proportion estimates. This calculation assumes PFS/OS proportion estimates of 50% to conservatively maximize the width of the obtained confidence intervals. Due to cohort size limitations multivariable modeling was not performed. All *p*‐values were two‐sided with statistical significance evaluated at the .05 alpha level. Ninety‐five percent confidence intervals for median PFS/OS survival time and six‐month PFS were calculated to assess the precision of the obtained estimates. Mann–Whitney U tests were used to identify significant differences between diffusion and permeability histogram values. All analyses were performed in R Version 3.6.0 (R Foundation for Statistical Computing, Vienna, Austria).

## 3 RESULTS

### 3.1 Study population

Patient demographics are displayed in Table 1. Sixteen patients with rHGG were treated with ICI+ SBRT, of which 10 were WHO grade IV and 6 were WHO grade III. MGMT methylation, IDH1 and TERT mutations were present in 4 (25%), 3 (19%) and 10 (63%) patients, respectively. Twelve patients received concurrent chemoradiation following their initial resection, and underwent an average of 4.5 lines of therapy. Five patients received ICI monotherapy, of which two were WHO grade IV and three were WHO grade III. MGMT methylation and TERT mutations were present in one and two patients, respectively, with no patients in this cohort possessing IDH mutations. Four patients underwent concurrent chemoradiation following their initial resection and received an average of 4.5 lines of therapy.

**TABLE 1:** Demographic and clinical characteristics

Patient characteristics		ICI + SBRT (*n* = 16)	ICI (*n* = 5)
Gender (*n*)	Female	9	3
	Male	7	2
Race (*n*)	Caucasian	13	4
	African American	1	0
	Other	2	1
Age (years)	Mean; Range	48.5; 22–81	56 ± 11; 26–67
KPS (*n*)	≥70	14	3
	<70	2	2
Initial resection extent (*n*)	Gross Total Resection	9	1
	Subtotal Resection	4	3
	Biopsy	3	1
WHO tumor grade (*n*)	4	10	2
	3	6	3
MGMT methylation status (*n*)	Methylated	4	1
	Unmethylated	12	4
IDH mutation status (*n*)	Mutated	3	0
	Wild type	0	5
TERT promotor mutation (*n*)	Mutated	10	2
	Wild type	0	3
Adjuvant TMZ + radiation (*n*)	Yes	12	4
	No	4	1
Lines of therapy (including TMZ + radiation)	Mean; Range	4.5 ± 1.7; 2–8	4.4 ± 1.6; 3–7

Abbreviations: IDH, isocitrate dehydrogenase; KPS, Karnofsky Performance Status; MGMT, O6‐Methylguanine‐DNA Methyltransferase; TERT, telomerase reverse transcriptase gene promoter; TMZ, temozolomide; WHO, World Health Organization.

On average, patients received three lines of therapy before they were offered ICI + SBRT (Table 2). SBRT doses ranged from 18 Gy in 1–3 fractions to 35 Gy in five fractions. A mean of 7.25 ICI cycles were given. Twelve patients received dexamethasone during their treatment, and six received bevacizumab during their treatment. Patients in the ICI monotherapy received an average of three prior lines of treatment. A mean of 6.2 ICI cycles were given. Three patients received dexamethasone and one was treated with bevacizumab. The average KPS at time of treatment initiation was 82 ± 10.8 and 72 ± 14 in the ICI + SBRT and ICI cohorts, respectively.

**TABLE 2:** Treatment data

Parameter		ICI + SBRT (*n* = 16)	ICI (*n* = 5)
ICI / ICI and SBRT given as what line of therapy (*n*)	Mean, range	4 ± 1.8; 2–8	4 ± 1.4; 2–5
Cycles ICI given	Mean, range	7.25; 2–22	6.2; 2–14
SBRT Dose (*n*)	35 Gy in five fractions	1	NA
	30 Gy in five fractions	9	
	27.5 Gy in five fractions	1	
	27 Gy in three fractions	2	
	25 Gy in five fractions	1	
	18 Gy in 1–3 fractions	2	
Average PFS from second line treatment onward	Mean, Range	4 ± 1.8; 2–8	8.6 ± 6.2; 3–17
Average KPS at time of intervention	Mean, Range	82 ± 10.8; 60–100	72 ± 14; 50–90
Average KPS at time of progression	Mean, Range	69 ± 18; 40–100	64 ± 13; 50–80
Average PFS on intervention	Mean, Range	4.1 ± 4; 1.2–14	8.6 ± 6.2; 3–17
Best one‐month response (RANO)	CR	0	0
	PR	2	0
	SD	8	2
	PD	6	3
6‐month PFS (*n*)	Progressed	13	4
	Stable	3	1
OS after intervention (months)	Mean, Range	7.75 ± 2.9; 4–16	13.8 ± 15; 2–38
Adverse events (CTCAE)	Grade 1	5	4
	Grade 2	1	0
	Grade ≥3	0	0
Steroids (*n*)	Yes	12	3
	No	4	2
Bevacizumab (*n*)	Yes	6	1
	No	10	4

Abbreviations: CR, complete response; CTCAE, common terminology criteria for adverse events; Gy, gray; ICI, immune checkpoint inhibition; KPS, Karnofsky performance status; NA, not applicable; OS, overall survival, RANO, response assessment in neuro‐oncology; PD, progressive disease; PFS, progression‐free survival; PR, partial response; SBRT, stereotactic body radiation therapy; SD, stable disease.

### 3.2 Adverse events

Among the ICI + SBRT cohort, there were four instances of grade 1 fatigue and one instance of grade 1 thrombocytopenia (Table 3). There was one instance of grade 2 fatigue, two instances each of grade 1 fatigue and grade 1 constipation among the ICI monotherapy cohort. There were no grade 3–5 AE, radiographic findings consistent with radiation necrosis on follow‐up imaging, or treatment‐related deaths in either cohort. No patients discontinued ICI due to toxicity.

**TABLE 3:** ICI monotherapy and ICI + SBRT toxicity in recurrent high‐grade gliomas

	ICI + SBRT					ICI monotherapy				
Toxicity	Grade 1	Grade 2	Grade 3	Grade 4	Total	Grade 1	Grade 2	Grade 3	Grade 4	Total
Anemia	0	0	0	0	0	0	0	0	0	0
Colitis	0	0	0	0	0	0	0	0	0	0
Constipation	0	0	0	0	0	2	0	0	0	
Fatigue	4	1	0	0	5	2	0	0	0	0
Intracranial hemorrhage	0	0	0	0	0	0	0	0	0	0
Hypertension	0	0	0	0	0	0	0	0	0	0
Infection without neutropenia	0	0	0	0	0	0	0	0	0	0
Lymphopenia	0	0	0	0	0	0	0	0	0	0
Nausea	0	0	0	0	0	0	0	0	0	0
Neutropenia	0	0	0	0	0	0	0	0	0	0
Pneumonitis	0	0	0	0	0	0	0	0	0	0
Thrombophlebitis	0	0	0	0	0	0	0	0	0	0
Thrombocytopenia	1	0	0	0	0	0	0	0	0	0
Wound Dehiscence	0	0	0	0	0	0	0	0	0	0
Totals	5	1	0	0	6	4	0	0	0	4

Abbreviations: ICI, immune checkpoint inhibition; SBRT, stereotactic body radiotherapy.

### 3.3 Response

**FIGURE 1:**
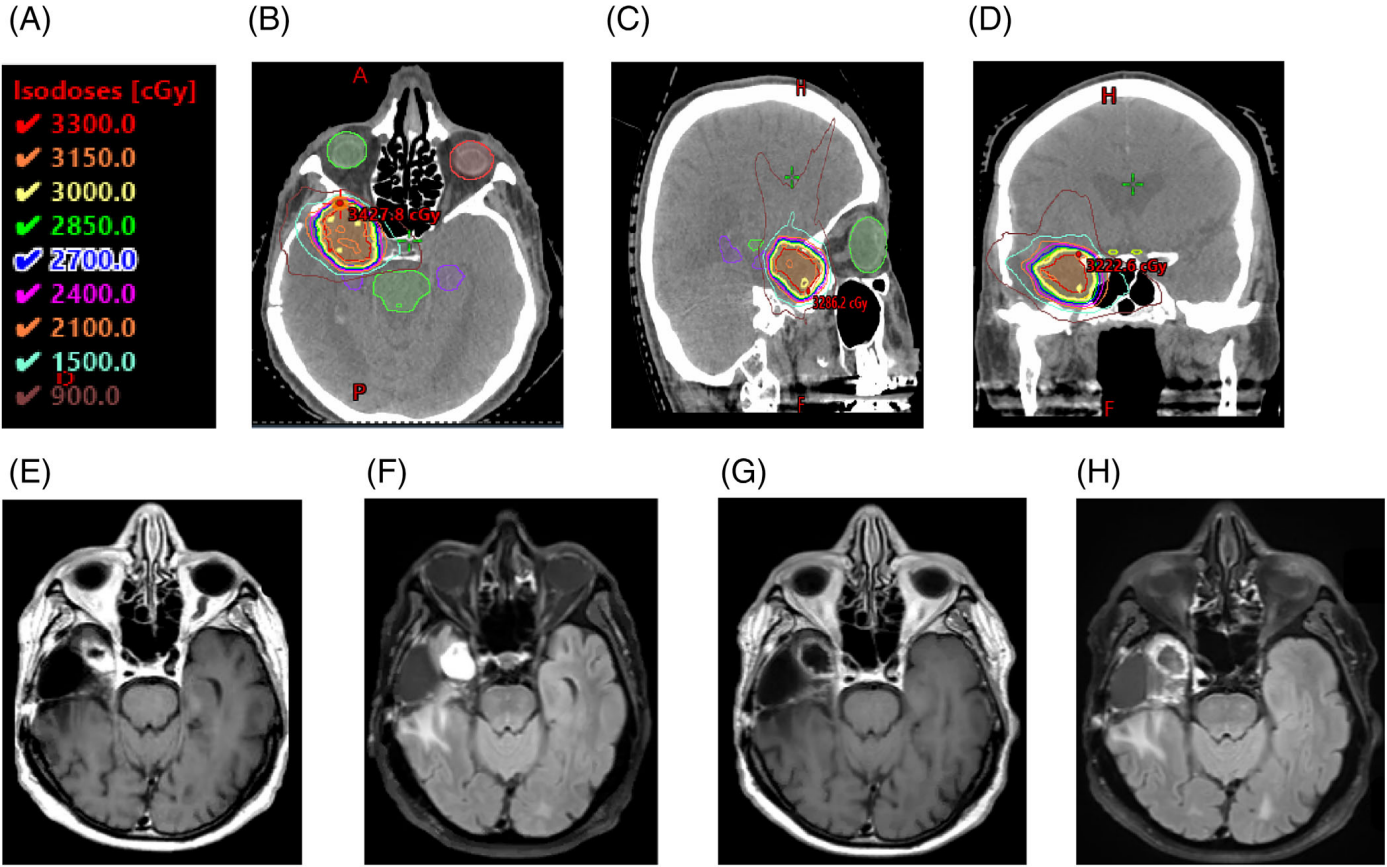
Partial Response in Patient Receiving ICI + SBRT. 83‐year‐old man with right temporal WHO grade IV glioblastoma status‐post resection and adjuvant concurrent radiotherapy and temozolomide who received 30 Gy in five fractions concurrently with 15 cycles of ICI as his third line treatment. Radiotherapy isodose line key shown in (A). Representative (B) axial (C) sagittal and (D) coronal images of his RT plan. Pretreatment MRI (E) axial T1 and (F) axial T2 FLAIR showing 2 × 2.1 cm nodular enhancing mass along anterior/medial margin of the resection cavity. MRI 4 months post‐SBRT (G) axial T1 and (H) axial T2 FLAIR showing overall decrease in size and nodular enhancing component of the lesion.

**FIGURE 2:**
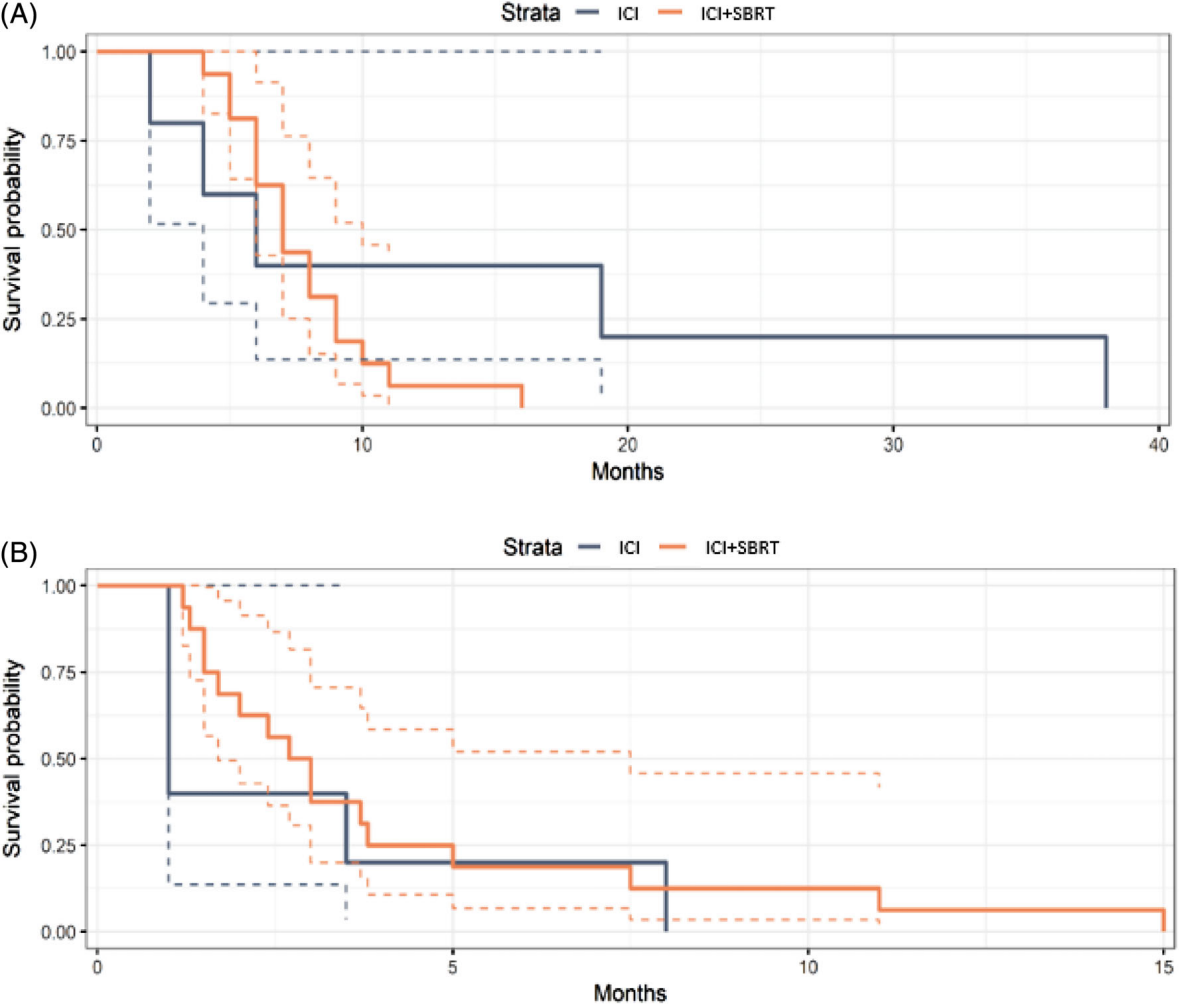
Kaplan–Meier curves for (A) progression free survival and (B) overall survival time. Dashed lines are 95% confidence intervals. Patients were followed until date of death (number at risk: 0).

Among the ICI + SBRT cohort, there were no CR, 2 PR, 8 SD and 6 PD at one month (Figure 1). Thirteen patients progressed at 6 months. The ICI monotherapy cohort had no CR or PR, 2 SD and 3 PD at one month, with 4 patients progressing by 6 months. The average KPS at time of progression was 69 ± 18 and 64 ± 13 in the ICI + SBRT and ICI cohorts, respectively. Patients were followed until date of death, with an average survival of 13.8 ± 15 months and 7.75 ± 2.9 months among the ICI and ICI + SBRT cohorts, respectively There were no significant differences in PFS (*p* = .4), OS (*p* = .3) or in median PFS time for patients receiving ICI + SBRT (2.85 months; 95% CI: 1.7, 7.5) or ICI (1 month; 95% CI: 1–unknown). Estimated six‐month PFS probability was 0.19 (95% CI: 0.07 to 0.52) for patients receiving ICI+ SBRT and 0.2 (95% CI: 0.035–1) for patients receiving ICI. The median OS was 7 months (95% CI: 6–10) for patients receiving ICI + SBRT and 6 months (95% CI: 4–unknown) for patients receiving ICI. Estimated six‐month OS probability was 0.625 (95% CI: 0.43–0.91) for patients receiving ICI + SBRT and 0.4 (95% CI: 0.14–1) for patients receiving ICI. Kaplan–Meier plots for PFS and OS are shown in Figure 2.

### 3.4 Radiologic analyses

DCE perfusion data was available in 15 of 16 ICI + SBRT patients pretreatment, 16 of 16 ICI+ SBRT patients posttreatment, and 5 of 5 ICI monotherapy patients pre and posttreatment. ADC maps were available in all 21 patients pre and posttreatment. There was a significant difference in tumor volumes pre and posttreatment (12.35 vs. 20.51; *p =* .03). However, no statistically significant difference was found in the other imaging metrics (reported pre vs. posttreatment): mean ADC (1.73 vs. 1.69; *p =* .58), mean Vp (6.63 vs. 5.78; *p =* .43); mean Ktrans (19.19 vs. 23.25; *p =* .63). A post hoc analysis comparing perfusion and diffusion imaging metrics between the ICI + SBRT and ICI monotherapy groups pre and posttreatment demonstrated no statistically significant difference in imaging metrics after drug initiation (reported pretreatment ICI + SBRT to posttreatment ICI + SBRT versus pretreatment ICI to posttreatment ICI): mean ADC (1.73–1.74 vs. 1.73–1.51; *p* = .18), mean Vp (7.39–6.04 vs. 4.35–4.98; *p* = .80), mean Ktrans (20.64–25.85 vs. 14.85–14.93; *p* = .46), tumor volume (13.25 vs. 22.33 vs. 8.74–13.20; *p* = .61).

## 4 DISCUSSION

There is a paucity of therapeutic options and no validated standard of care for rHGG. While awaiting results of ongoing prospective trials (Table 4), this retrospective series demonstrated ICI with concurrent re‐irradiation using SBRT can be safely delivered in rHGG. Preclinical data provides a rationale for evaluating ICI in this clinical setting.^20^, ^24^ The statistically significant increase in posttreatment tumor volumes identified within our cohort may in part have reflected a component of pseudoprogression related to ICI. Prior studies associate ICI treatment response with a preceding increase in tumor volume related to intratumoral immune cell infiltration, resulting in a transient inflammatory reaction.^25^

**TABLE 4:** Ongoing clinical trials for ICI + SBRT for recurrent gliomas

Agent	*N*	Experimental design	Reference
Nivolumab	17	SBRT + nivolumab + valproate	NCT02648633
Nivolumab and Ipilimumab	33	SBRT + nivolumab, ipilimumab and bevacizumab	NCT02829931
Pembrolizumab	32	SBRT + Pembrolizumab and Bevacizumab	NCT02313272
Durvalumab	62	Durvalumab + SBRT versus SBRT	NCT02866747

Abbreviations: ICI, immune checkpoint inhibition; SBRT, stereotactic body radiotherapy.

Understanding ICI response requires further elucidation of the intratumoral milieu and systemic immune response.^26^ Several studies support preoperative ICI enhance expression of chemokine transcripts including interferon‐γ, increase immune cell infiltration, and augment T cell receptor clonal variety, but with conflicting clinical results.^27^, ^28^ One putative explanation for suboptimal immune response is the high rate of lymphopenia observed in HGG patients, with one group reporting that T cells are available in this population but are sequestered in the bone marrow.^29^

The aforementioned studies provide a rationale for optimizing immunotherapeutic efficacy through its implementation in an immunologically favorable setting, such as priming the immune system to tumor‐specific antigens. RT may improve ICI effects by increasing the availability and diversity of intracellular peptides, increasing MHC class I expression, and promoting T cell recruitment and infiltration.^20^, ^21^, ^30^, ^31^ Technological advancements in the delivery of SBRT allow for highly conformal treatments that substantially reduce AE associated with re‐irradiation in other disease sites.^16^ Several studies show function status improvements and decreased reliance on corticosteroid following SBRT monotherapy with a low risk of late central nervous system toxicity.^14^, ^15^, ^17^, ^18^, ^32^ Additionally, SBRT dose‐fractionation schemes may be more effective than conventionally fractionated RT with regard to augmenting immune responses.^21^, ^33^ This option also allows RT completion within one to five treatments, which is convenient for patients.

A closer look at this cohort notes several limitations that could be considered in future studies geared towards optimizing a response. Most patients had several recurrences and subsequently received multiple systemic therapies either on or off clinical trials. There was a mean of 4.5 lines of treatment administered with ICI + SBRT therapy given as the last line in 8 of these patients. There is a possibility that these prior treatments negatively impacted the ability to stimulate immune responses, and more robust responses may be seen if treated with ICI + SBRT at first recurrence. Patients were treated without knowing PD‐L1 expression status. A few reports show higher response rates with increased expression in other malignancies,^34^ however the prognostic value of PD‐L1 for HGG is still under investigation. While foundational analyses were available, advanced correlation studies were limited by the cohort size. Two‐thirds of the patients in this cohort were on dexamethasone while receiving ICI which may interfere with the ICI efficacy.

The optimal treatment approach for patients with rHGG continues to be an area of ongoing investigation. This small retrospective study suggests ICI can be safely given concurrently with re‐irradiation using SBRT for patients with rHGG. These initial findings support evaluating whether optimizing conditions for combinatory ICI + SBRT approaches may lead to favorable clinical responses, or whether attention should be turned to other therapeutic avenues to address this unmet need in neuro‐oncology.

## AUTHOR CONTRIBUTIONS

**Sean S. Mahase:** Conceptualization (lead); data curation (lead); investigation (lead); project administration (lead); writing – original draft (lead); writing – review and editing (lead). **Michelle Roytman:** Data curation (equal); investigation (equal); writing – original draft (equal). **Diana Roth O’Brien:** Data curation (equal); investigation (equal); writing – original draft (equal). **Jana Ivanidze:** Formal analysis (equal); investigation (equal); supervision (equal); writing – review and editing (equal). **Theodore H. Schwartz:** Writing – review and editing (equal). **Susan C. Pannullo:** Writing – review and editing (equal). **Rohan Ramakrishna:** Writing – review and editing (equal). **Rajiv S. Magge:** Writing – review and editing (equal). **Nicholas Williams:** Formal analysis (equal); methodology (equal); software (equal); validation (equal). **Howard A. Fine:** Writing – review and editing (equal). **Gloria Chia‐Yi Chiang:** Conceptualization (lead); investigation (equal); methodology (lead); project administration (equal); supervision (lead); writing – original draft (equal); writing – review and editing (lead). **Jonathan PS Knisely:** Conceptualization (lead); investigation (lead); methodology (lead); project administration (lead); software (equal); supervision (lead); writing – original draft (lead); writing – review and editing (lead).

## FUNDING INFORMATION

The authors did not receive support from any organization for the submitted work.

## CONFLICTS OF INTEREST STATEMENT

The authors have no relevant financial or nonfinancial interests to disclose.

### ETHICS STATEMENT

This retrospective chart review study involving human participants was in accordance with the ethical standards of the institutional and national research committee and with the 1964 Helsinki Declaration and its later amendments or comparable ethical standards. The Human Investigation Committee (IRB) of Weill Cornell Medicine approved this study (19–07020426) .

## Supporting information


**Supplementary Figure 1:** Flow diagram of study participants screened for analysis. ICI, immune checkpoint inhibition; SBRT, stereotactic body radiotherapy

## Data Availability

The datasets generated during and/or analyzed during the current study are available from the corresponding author on reasonable request.

## References

[1] Cairncross G , Wang M , Shaw E , et al. Phase III trial of Chemoradiotherapy for anaplastic Oligodendroglioma: long‐term results of RTOG 9402. J Clin Oncol. 2013;31(3):337‐343. 23071247 10.1200/JCO.2012.43.2674 PMC3732012

[2] Chang S , Zhang P , Cairncross JG , et al. Phase III randomized study of radiation and temozolomide versus radiation and nitrosourea therapy for anaplastic astrocytoma: results of NRG oncology RTOG 9813. Neuro Oncol. 2017;19(2):252‐258. 27994066 10.1093/neuonc/now236 PMC5463834

[3] Stupp R , Hegi ME , Mason WP , et al. Effects of radiotherapy with concomitant and adjuvant temozolomide versus radiotherapy alone on survival in glioblastoma in a randomised phase III study: 5‐year analysis of the EORTC‐NCIC trial. Lancet Oncol. 2009;10(5):459‐466. 19269895 10.1016/S1470-2045(09)70025-7

[4] Stupp R , Taillibert S , Kanner A , et al. Effect of tumor‐treating fields plus maintenance Temozolomide vs maintenance Temozolomide alone on survival in patients with glioblastoma: a randomized clinical trial. Jama. 2017;318(23):2306‐2316. 29260225 10.1001/jama.2017.18718 PMC5820703

[5] van den Bent MJ , Baumert B , Erridge SC , et al. Interim results from the CATNON trial (EORTC study 26053‐22054) of treatment with concurrent and adjuvant temozolomide for 1p/19q non‐co‐deleted anaplastic glioma: a phase 3, randomised, open‐label intergroup study. Lancet. 2017;390(10103):1645‐1653. 28801186 10.1016/S0140-6736(17)31442-3 PMC5806535

[6] Norden AD , Young GS , Setayesh K , et al. Bevacizumab for recurrent malignant gliomas: efficacy, toxicity, and patterns of recurrence. Neurology. 2008;70(10):779‐787. 18316689 10.1212/01.wnl.0000304121.57857.38

[7] Taal W , Oosterkamp HM , Walenkamp AM , et al. Single‐agent bevacizumab or lomustine versus a combination of bevacizumab plus lomustine in patients with recurrent glioblastoma (BELOB trial): a randomised controlled phase 2 trial. Lancet Oncol. 2014;15(9):943‐953. 25035291 10.1016/S1470-2045(14)70314-6

[8] Suchorska B , Weller M , Tabatabai G , et al. Complete resection of contrast‐enhancing tumor volume is associated with improved survival in recurrent glioblastoma‐results from the DIRECTOR trial. Neuro Oncol. 2016;18(4):549‐556. 26823503 10.1093/neuonc/nov326 PMC4799687

[9] Chiocca EA , Nassiri F , Wang J , Peruzzi P , Zadeh G . Viral and other therapies for recurrent glioblastoma: is a 24‐month durable response unusual? Neuro Oncol. 2019;21(1):14‐25. 30346600 10.1093/neuonc/noy170 PMC6303472

[10] Kamiya‐Matsuoka C , Gilbert MR . Treating recurrent glioblastoma: an update. CNS Oncol. 2015;4(2):91‐104. 25768333 10.2217/cns.14.55 PMC6093021

[11] Tsien CI , Pugh SL , Dicker AP , et al. NRG oncology/RTOG1205: a randomized phase II trial of concurrent bevacizumab and Reirradiation versus bevacizumab alone as treatment for recurrent glioblastoma. J Clin Oncol. 2022:JCO2200164. Online ahead of print. 10.1200/JCO.22.00164 PMC9940937 36260832

[12] Naidoo J , Page DB , Wolchok JD . Immune modulation for cancer therapy. Br J Cancer. 2014;111(12):2214‐2219. 25211661 10.1038/bjc.2014.348 PMC4264429

[13] Goldberg SB , Gettinger SN , Mahajan A , et al. Pembrolizumab for patients with melanoma or non‐small‐cell lung cancer and untreated brain metastases: early analysis of a non‐randomised, open‐label, phase 2 trial. Lancet Oncol. 2016;17(7):976‐983. 27267608 10.1016/S1470-2045(16)30053-5 PMC5526047

[14] Combs SE , Thilmann C , Edler L , Debus J , Schulz‐Ertner D . Efficacy of fractionated stereotactic reirradiation in recurrent gliomas: long‐term results in 172 patients treated in a single institution. J Clin Oncol. 2005;23(34):8863‐8869. 16314646 10.1200/JCO.2005.03.4157

[15] Nieder C , Astner ST , Mehta MP , Grosu AL , Molls M . Improvement, clinical course, and quality of life after palliative radiotherapy for recurrent glioblastoma. Am J Clin Oncol. 2008;31(3):300‐305. 18525311 10.1097/COC.0b013e31815e3fdc

[16] Fogh SE , Andrews DW , Glass J , et al. Hypofractionated stereotactic radiation therapy: an effective therapy for recurrent high‐grade gliomas. J Clin Oncol. 2010;28(18):3048‐3053. 20479391 10.1200/JCO.2009.25.6941 PMC2982785

[17] Niyazi M , Sohn M , Schwarz SB , Lang P , Belka C , Ganswindt U . Radiation treatment parameters for re‐irradiation of malignant glioma. Strahlenther Onkol. 2012;188(4):328‐333. 22349710 10.1007/s00066-011-0055-2

[18] Hudes RS , Corn BW , Werner‐Wasik M , et al. A phase I dose escalation study of hypofractionated stereotactic radiotherapy as salvage therapy for persistent or recurrent malignant glioma. Int J Radiat Oncol Biol Phys. 1999;43(2):293‐298. 10030252 10.1016/s0360-3016(98)00416-7

[19] Lugade AA , Sorensen EW , Gerber SA , Moran JP , Frelinger JG , Lord EM . Radiation‐induced IFN‐gamma production within the tumor microenvironment influences antitumor immunity. J Immunol. 2008;180(5):3132‐3139. 18292536 10.4049/jimmunol.180.5.3132

[20] Zeng J , See AP , Phallen J , et al. Anti‐PD‐1 blockade and stereotactic radiation produce long‐term survival in mice with intracranial gliomas. Int J Radiat Oncol Biol Phys. 2013;86(2):343‐349. 23462419 10.1016/j.ijrobp.2012.12.025 PMC3963403

[21] Vanpouille‐Box C , Alard A , Aryankalayil MJ , et al. DNA exonuclease Trex1 regulates radiotherapy‐induced tumour immunogenicity. Nat Commun. 2017;8:15618. 28598415 10.1038/ncomms15618 PMC5472757

[22] Ivanidze J , Lum M , Pisapia D , et al. MRI features associated with TERT promoter mutation status in glioblastoma. J Neuroimaging. 2019;29(3):357‐363. 30644143 10.1111/jon.12596

[23] Wen PY , Macdonald DR , Reardon DA , et al. Updated response assessment criteria for high‐grade gliomas: response assessment in neuro‐oncology working group. J Clin Oncol. 2010;28(11):1963‐1972. 20231676 10.1200/JCO.2009.26.3541

[24] Wainwright DA , Chang AL , Dey M , et al. Durable therapeutic efficacy utilizing combinatorial blockade against IDO, CTLA‐4, and PD‐L1 in mice with brain tumors. Clin Cancer Res. 2014;20(20):5290‐5301. 24691018 10.1158/1078-0432.CCR-14-0514 PMC4182350

[25] Onesti CE , Freres P , Jerusalem G . Atypical patterns of response to immune checkpoint inhibitors: interpreting pseudoprogression and hyperprogression in decision making for patients' treatment. J Thorac Dis. 2019;11(1):35‐38. 30863564 10.21037/jtd.2018.12.47 PMC6384391

[26] Waziri A . Glioblastoma‐derived mechanisms of systemic immunosuppression. Neurosurg Clin N Am. 2010;21(1):31‐42. 19944964 10.1016/j.nec.2009.08.005

[27] Schalper KA , Rodriguez‐Ruiz ME , Diez‐Valle R , et al. Neoadjuvant nivolumab modifies the tumor immune microenvironment in resectable glioblastoma. Nat Med. 2019;25(3):470‐476. 30742120 10.1038/s41591-018-0339-5

[28] Cloughesy TF , Mochizuki AY , Orpilla JR , et al. Neoadjuvant anti‐PD‐1 immunotherapy promotes a survival benefit with intratumoral and systemic immune responses in recurrent glioblastoma. Nat Med. 2019;25(3):477‐486. 30742122 10.1038/s41591-018-0337-7 PMC6408961

[29] Chongsathidkiet P , Jackson C , Koyama S , et al. Sequestration of T cells in bone marrow in the setting of glioblastoma and other intracranial tumors. Nat Med. 2018;24(9):1459‐1468. 30104766 10.1038/s41591-018-0135-2 PMC6129206

[30] Burnette BC , Liang H , Lee Y , et al. The efficacy of radiotherapy relies upon induction of type i interferon‐dependent innate and adaptive immunity. Cancer Res. 2011;71(7):2488‐2496. 21300764 10.1158/0008-5472.CAN-10-2820 PMC3070872

[31] Newcomb EW , Demaria S , Lukyanov Y , et al. The combination of ionizing radiation and peripheral vaccination produces long‐term survival of mice bearing established invasive GL261 gliomas. Clin Cancer Res. 2006;12(15):4730‐4737. 16899624 10.1158/1078-0432.CCR-06-0593

[32] Laing RW , Warrington AP , Graham J , Britton J , Hines F , Brada M . Efficacy and toxicity of fractionated stereotactic radiotherapy in the treatment of recurrent gliomas (phase I/II study). Radiother Oncol. 1993;27(1):22‐29. 8327729 10.1016/0167-8140(93)90040-f

[33] Hwang WL , Pike LRG , Royce TJ , Mahal BA , Loeffler JS . Safety of combining radiotherapy with immune‐checkpoint inhibition. Nat Rev Clin Oncol. 2018;15(8):477‐494. 29872177 10.1038/s41571-018-0046-7

[34] Herbst RS , Soria JC , Kowanetz M , et al. Predictive correlates of response to the anti‐PD‐L1 antibody MPDL3280A in cancer patients. Nature. 2014;515(7528):563‐567. 25428504 10.1038/nature14011 PMC4836193

