# Supplementary material for: Concurrent immunotherapy and re‐irradiation utilizing stereotactic body radiotherapy for recurrent high‐grade gliomas
Source: Cancer Rep (Hoboken). 2023 Feb 7;6(7):e1788. doi: 10.1002/cnr2.1788 (PMC10363830; doi:10.1002/cnr2.1788)
Supplement: Supplementary file 1 — Supplementary Figure 1: Flow diagram of study participants screened for analysis. ICI, immune checkpoint inhibition; SBRT, stereotactic body radiotherapy [file CNR2-6-e1788-s001.pdf]

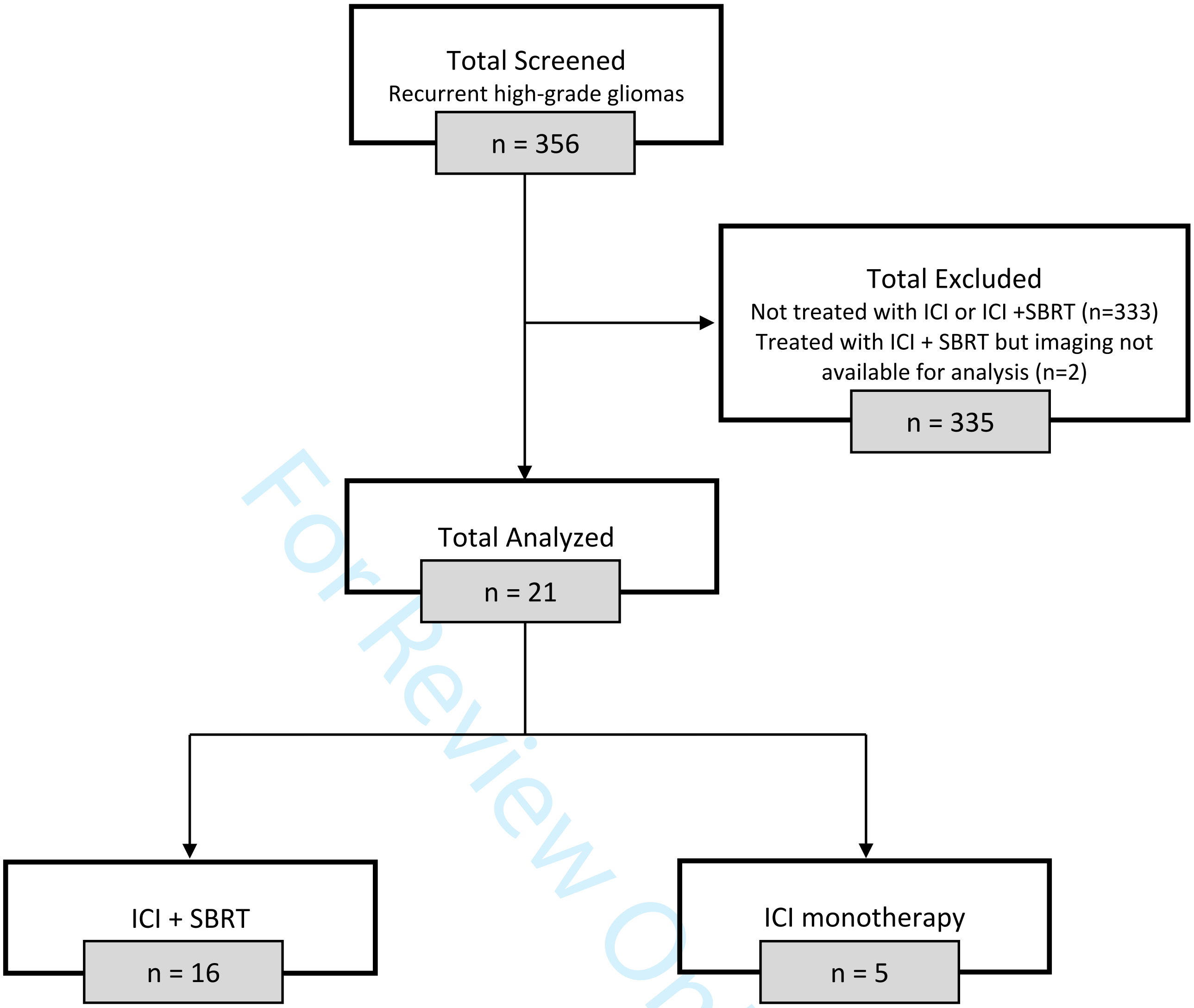

For Review Only

Supplementary Fig. 1: Flow diagram of study participants screened for analysis. *Abbreviations*: ICI: immune checkpoint inhibition; SBRT: stereotactic body radiotherapy
